# Association between Asymptomatic Hyperuricemia with Adiposity Indices: A Cross-Sectional Study in a Spanish Population

**DOI:** 10.3390/nu15224798

**Published:** 2023-11-16

**Authors:** Carmen Sánchez-Bacaicoa, Esperanza Santano-Mogena, Sergio Rico-Martín, Purificación Rey-Sánchez, Raúl Juárez-Vela, Juan F. Sánchez Muñoz-Torrero, Fidel López-Espuela, Julián F. Calderón-García

**Affiliations:** 1 Department of Family Medicine, Hospital of Menorca, 07703 Menorca, Spain; carmensbacaicoa@gmail.com; 2 Department of Nursing, Nursing and Occupational Therapy College, University of Extremadura, 10003 Cáceres, Spain; esantano@unex.es (E.S.-M.); prey@unex.es (P.R.-S.); fidellopez@unex.es (F.L.-E.); jfcalgar@unex.es (J.F.C.-G.); 3 Department of Nursing, Faculty of Health Sciences, University of La Rioja, 26006 Logroño, Spain; raul.juarez@unirioja.es; 4 Department of Internal Medicine, Hospital San Pedro Alcántara, 10003 Cáceres, Spain; juanf.sanchezm@gmail.com

**Keywords:** anthropometric indices, hyperuricemia, cardiovascular risk factors, cardiovascular disease, obesity

## Abstract

Introduction: New anthropometric indices have been developed as an alternative to body mass index (BMI) and waist circumference (WC) to assess body mass and visceral fat. Asymptomatic hyperuricemia is considered an independent cardiovascular risk factor. Currently, little is known about the relationship between asymptomatic hyperuricemia and several new anthropometric indices. This study aimed to assess the association between the presence of asymptomatic hyperuricemia and anthropometric indices, both novel and traditional. Methods: This study analyzed 1094 Spanish subjects who consecutively visited the cardiovascular risk consultation of the University Hospital San Pedro de Alcántara of Cáceres, Spain, between June 2021 and September 2022. Anthropometric measures, including traditional and novel indices, were determined. The asymptomatic hyperuricemia group was defined according to serum uric acid levels. Results: All the anthropometric indices studied, including new and traditional, were significantly greater among patients with asymptomatic hyperuricemia, except for WWI. In multiple linear regression analysis, serum uric acid levels were significantly correlated with BMI, WHR, WHtR, AVI, BAI, BRI, CUN-BAE, and WWI but not ABSI or CI. In the univariate analysis, all indices were associated with asymptomatic hyperuricemia (*p* < 0.05); however, only WHtR (adjusted OR: 2.93; 95% CI: 1.03–8.37; *p* = 0.044), AVI (adjusted OR: 1.46; 95% CI: 1.04–2.04; *p* = 0.026), and BRI (adjusted OR: 1.66; 95% CI: 1.19–2.32; *p* = 0.003) were significantly associated in multivariate analysis. Finally, WHtR, AVI, and BRI provided the largest AUCs. Conclusions: Our findings showed that WHtR, AVI, and BRI were independently positively associated with asymptomatic hyperuricemia and could be good predictors.

## 1. Introduction

Uric acid, a product of purine metabolism, is one of the main endogenous antioxidants in organisms and regulates several biological processes [1]. However, dietary and genetic factors can lead to changes in uric acid metabolism, increasing uric acid levels [2]. Excess intake of purines (usually includes intakes high in fats and sugars) is generally the major cause of elevated serum uric acid levels in individuals with normal kidney function [3]. Hyperuricemia is defined as the presence of elevated serum uric acid levels (≥7 mg/dL in males and ≥6 mg/dL in females) [4]. This pathology is traditionally associated with gout; however, approximately two-thirds or more of such individuals remain asymptomatic, never developing gout, uric acid nephrolithiasis, or hyperuricemia nephropathy [5], although these clinical manifestations may develop in hyperuricemic individuals at any point. Persistent hyperuricemia is considered an independent risk factor for hypertension [6,7], diabetes [8], metabolic syndrome [9], and cardiovascular events [10], including stroke [11], coronary heart disease [12], and peripheral artery disease [13].

Obesity, defined as excessive accumulation of body fat, may play an important role in the development of hyperuricemia and vice versa [14]. Traditional anthropometric indices, including body mass index (BMI), waist-to-hip ratio (WHR), and waist-to-height ratio (WHtR), are the most widely used in daily clinical practice [15]. BMI is the main anthropometric index used to stratify patients as overweight or obese [16]; however, BMI does not differentiate between fat and lean mass and does not differentiate between visceral or subcutaneous adiposity [17]. In addition, although cardiovascular risk is increased by obesity categorized by BMI, some studies have described the phenomenon of the “obesity paradox”, where overweight or obese individuals may have a better prognosis than individuals with normal weight [18,19,20]. Waist circumference (WC), WHR, and WHtR were proposed as central obesity indicators for their relationship with adiposity distribution [21,22,23]. The main limitation of WC is that it does not consider the individual’s weight and height [24]. WHR measurement offers no benefit over WC alone and is not recommended as part of the routine obesity evaluation [15]; therefore, clinicians rarely use it. On the other hand, WHtR has been shown to be statistically more important than WHR, WC, and BMI WC in predicting cardiovascular abnormalities [23]. However, measuring height in addition to WC provides no extra advantage [25].

Alternatively, new anthropometric indices have been proposed to identify central and visceral adiposity [26] due to the limitations in the measurement and distribution of body fat that traditional anthropometric indices present [17,24,25]. All new anthropometric indices assess general and visceral obesity and have been related to cardiovascular disorders and established vascular disease, including BMI [26,27,28,29,30,31]. The most researched anthropometric indices are the body adiposity index (ABSI) [32], abdominal volume index (AVI) [33], body adiposity index (BAI) [34], body roundness index (BRI) [35], conicity index (CI) [36], Clinica Universitaria de Navarra—Body adiposity estimator (CUN-BAE) [37], and weight-adjusted waist index (WWI) [38].

At present, few studies have been published assessing the association of any of the new anthropometric indices with hyperuricemia [39,40,41,42]. None of the studies have been conducted on a European population. The purpose of this study was to investigate which anthropometric indices are best associated with hyperuricemia in a Spanish population.

## 2. Methods

An observational cross-sectional study was conducted between June 2021 and September 2022. It included 1094 participants who visited the Cardiovascular Risk consultation of the GEEVAS (Grupo de Estudios de Enfermedades VASculares) research group of the University Hospital San Pedro de Alcántara of Cáceres, Spain. Our intent was to have enough statistical power to identify low effect sizes (anticipated Cohen’s δ = 0.20) with α = 0.05 and β = 0.95, which required a minimum sample size of 1084 subjects.

**Figure 1:**
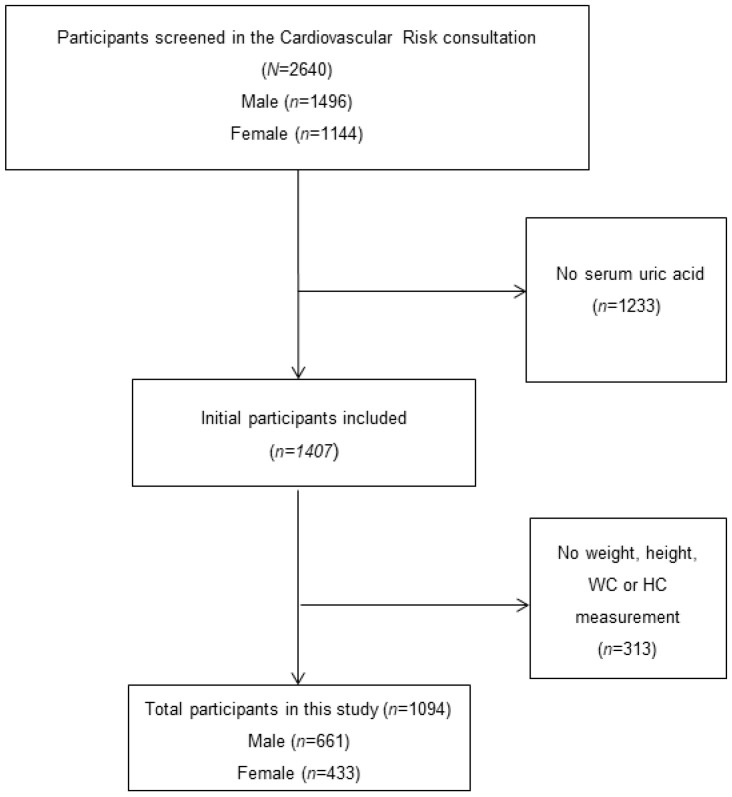
Participant selection process.

Figure 1 shows the subject selection process for this study. Briefly, 2640 patients were screened (of whom 1233 were excluded due to no present serum acid uric collection). A total of 1407 subjects were recruited; however, 313 were excluded because no anthropometric measurements were carried out. There were a total of 1094 adults with complete data in this study. We excluded symptomatic hyperuricemia subjects, pregnant women, and participants with previously confirmed severe medical diseases such as mental disorders, cancer, or terminal disease diagnosis. Written consent was obtained from all participants after they were previously informed of the purpose of the investigation. The study protocol was designed in accordance with the Declaration of Helsinki, and ethical approval was granted from the Ethics Committee (Ref. 047-2021) of the University Hospital San Pedro de Alcántara of Cáceres, Spain.

### 2.1. Study Variable and Definitions

Clinical variables related to vascular risk (age, male sex, sedentary lifestyle, current smoking status, presence of diabetes, hypertension, dyslipidemia, obesity, and metabolic syndrome), previous cardiovascular events, and current medical treatment were obtained from the patient’s medical history. The subjects’ blood samples were collected after a minimum of ten hours of overnight fasting. Serum levels of uric acid, total cholesterol (TC), low-density lipoprotein cholesterol (LDL), triglycerides, fasting plasma glucose (FPG), and glycated hemoglobin (HbA1c) were determined using a biochemical autoanalyzer in the biochemistry laboratory of the University Hospital San Pedro de Alcántara of Cáceres, Spain. Asymptomatic hyperuricemia was defined as serum uric acid levels ≥7 mg/dL in men or ≥6 mg/dL in women and no presence of signs and symptoms of monosodium urate crystal deposition disease, such as gout or uric acid renal disease [4].

A physical assessment was carried out on each participant. Blood pressure (BP) measurement was performed with an oscillometric device (OMRON model HEM-907) following the European Society of Hypertension recommendations [43]. Systolic (SBP) and diastolic blood pressure (DBP) values were calculated as the mean of the last two measurements of a total of three performed early in the morning with the subject relaxed and seated. Afterward, we calculated the pulse pressure (PP): PP = SBP − DBP.

Metabolic syndrome was defined according to the most recent Joint Interim Statement of the International Diabetes Federation [44] as the presence of three or more of the following five components: FPG ≥ 100 mg/dL or antidiabetic drug treatment; high-density lipoprotein (HDL) cholesterol <40 mg/dL in males or <50 mg/dL in females; triglycerides ≥150 mg/dL or drug treatment for elevated triglycerides; SBP ≥ 130 mmHg or DBP ≥ 85 mmHg or antihypertensive drug treatment and abdominal obesity in European population according to European guidelines on cardiovascular disease [45] (WC ≥ 102 cm in males and ≥88 cm in females. 

Body weight and height were obtained with participants barefoot and wearing light clothing. BMI was calculated as weight (kg)/height^2^ (m^2^). Hip circumference (HC) and waist circumference (WC) were measured with a nonelastic tape according to the Spanish Society for the Study of Obesity recommendations [46]. WHR was determined as the ratio of WC (cm) to HC (cm), and WHtR was determined as the ratio of WC (cm) to height (cm).

Finally, novel adiposity indices were calculated using the following formulas:

$$ABSI=\frac{WC\;\left(m\right)}{{BMI}^{\frac{2}{3}}\;\left(kg/m^{2}\right)\cdot {Height}^{\frac{1}{2}}\;\left(m\right)}$$

$$AVI=\frac{2\cdot {WC}^{2}\left(cm\right)+{0.7\;\left(WC-HC\right)}^{2}\;\left(cm\right)}{1000}$$

$$BAI=\frac{HC\;\left(m\right)}{{Height}^{\frac{2}{3}}\;\left(m\right)}-18$$

$$BRI=364.2-365.52\cdot \sqrt{1-\left(\frac{{WC\;\left(m\right)/2\pi )}^{2}}{{\left(0.5\;height\;\left(m\right)\right)}^{2}}\right)}$$

$$CI=0.109^{-1}\cdot WC\left(m\right)\cdot {\left(\frac{Weight\left(kg\right)}{Height\left(m\right)}\right)}^{-\frac{1}{2}}$$

$$\begin{array}{ll} CUNBAE= & \left(3.1723\cdot BMI\right)-\left(0.026\cdot {BMI}^{2}\right)\\ & +\left(0.181\cdot BMI\cdot sex\right)-\left(0.02\cdot BMI\cdot age\right)\\ & -\left(0.005\cdot {BMI}^{2}\cdot sex\right)+(0.00021\;\cdot \;{BMI}^{2}\;\cdot \;age) \end{array}$$

where sex is 1 for female and 0 for male = 0. Age is measured in years.

$$WWI=\frac{WC\;(cm)}{\sqrt{weight\;(kg)}}$$

Abbreviations: ABSI, a body shape index; AVI, abdominal volume index; BAI, body adiposity index; BRI, body roundness index; CI, conicity index; CUNBAE, Clínica Universidad de Navarra-Body Adiposity Estimator; WWI, weight-adjusted waist index.

### 2.2. Statistical Analysis

Statistical analysis was performed using IBM SPSS version 27.0 (IBM Corporation, Armonk, NY, USA) software for Windows. Categorical variables are reported as frequencies (%), and continuous variables are reported as averages ± standard deviations. Participants were compared according to the absence or presence of asymptomatic hyperuricemia. For categorical variables, the χ^2^ or Fisher’s exact test was used (if the frequency observed in any of the groups was less than 5). The Student’s *t*-test (if distribution was normal) or the Mann-Whitney U test (if distribution was non-normal) was applied to compare continuous variables. A normal distribution was revealed when *p* > 0.05 was found by the Kolmogorov-Smirnov test.

Pearson’s correlation test was used to investigate the relationship between adiposity indices and serum uric acid levels. Moreover, multiple linear regression utilizing the enter method was used to determine whether the studied variables predicted high serum uric acid levels. The variables included in the modeling were age (years), SBP (mmHg), DBP (mmHg), PP (mmHg), total cholesterol (mg/dL), HDL (mg/dL), LDL (mg/dL), triglycerides (mg/dL), FPG (mg/dL), HbA1C (%).

On the other hand, univariate and multivariate logistic regression analyses were conducted to investigate the possible association between the dependent variable (presence of hyperuricemia) and independent variables. Due to the lack of cut-off points for the new anthropometric indices, the values of the greatest quartile were considered (ABSI ≥ 0.086; AVI ≥ 23.85; BAI ≥ 36.23; BRI ≥ 6.92; CI ≥ 1.39; CUN-BAE ≥ 41.07, and WWI ≥ 11.72), while traditional anthropometric measures and previously published pathological cut-off points were assumed (BMI≥ 30 kg/m^2^; WHR > 0.85 in women or 0.94 in men, and WHtR > 0.5). Odds ratios (ORs) and subsequent 95% confidence intervals (CIs) were calculated. Independent variables were included in the multivariate analysis when *p* < 0.10 was found in the univariate analysis.

The area under the curve (AUC) and the corresponding 95% confidence intervals (CIs) were calculated by receiver operating characteristic (ROC) analysis. The optimal cut-off values of indices to detect hyperuricemia from ROC analyses were analyzed by the maximum Youden’s index (sensitivity + specificity − 1).

## 3. Results

A total of 1094 (60.4% men) patients with a mean age of 54.77 ± 12.79 years were investigated. Of these, 232 (21.2%) had asymptomatic hyperuricemia. The variables were compared according to serum uric acid levels (Table 1). Significant differences were found between groups in age, systolic blood pressure, pulse pressure, total cholesterol, LDL-cholesterol, triglyceride, fast plasma glucose, and glycated hemoglobin, where individuals with asymptomatic hyperuricemia had higher values than those with normouricemia, except for total and LDL cholesterol, where subjects with hyperuricemia showed significantly lower values. Moreover, the asymptomatic hyperuricemia group had significantly more subjects with hypertension, diabetes, obesity, metabolic syndrome, previous cardiovascular events, and the use of antihypertensive and antidiabetic drugs. Regarding the anthropometric indices studied, both the traditional and the new indices presented significantly higher values in the asymptomatic hyperuricemia group.

On the other hand, serum uric acid levels were significantly correlated with all anthropometric indices studied (Table 2). We further analyzed the possible significant relationship of serum uric acid levels to the anthropometric indices studied by multiple linear regression, where we observed a positive relation with all the anthropometric indices except for ABSI and CI.

Table 3 reports the predictor variables that were significantly associated with the presence of asymptomatic hyperuricemia (univariate analysis). These were age ≥ 65 years, current smokers, hypertension, diabetes, metabolic syndrome, cardiovascular event, SBP ≥ 140 mmHg, PP ≥ 60 mmHg, TC ≥ 190 mg/dL, LDL ≥ 100 mg/dL, triglyceride ≥ 200 mg/dL, FPG ≥ 126 mg/dL, antihypertensive drugs, and antidiabetic drugs.

In the univariate analysis of anthropometric indices (Table 4), all of these presented a significantly positive association with asymptomatic hyperuricemia presence (*p* < 0.05). However, only WHtR (aOR: 2.93; 95% CI: 1.03–8.37; *p* = 0.044), AVI (aOR: 1.46; 95% CI: 1.04–2.04; *p* = 0.026), and BRI (aOR: 1.66; 95% CI: 1.19–2.32; *p* = 0.003) were significantly associated in the multivariate analysis.

**Figure 2:**
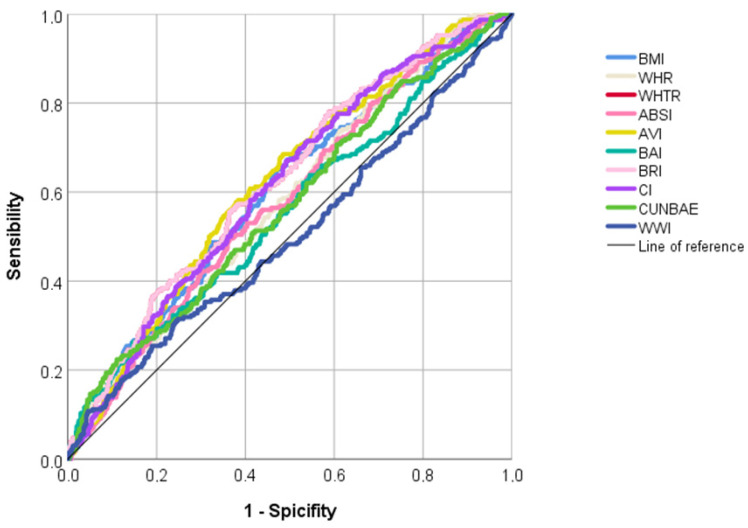
ROC analysis to predict asymptomatic hyperuricemia.

Finally, according to the ROC analyses (Table 5 and Figure 2), WHtR, AVI, and BRI showed the largest area under the curve (AUC: 0.624, 0.621, and 0.624, respectively), and WWI showed the smallest AUC (AUC: 0.503). The optimal cut-off values to detect hyperuricemia from ROC analyses were 0.57 for WHtR, 19.55 for AVI, and 5.97 for BRI.

## 4. Discussion

In this cross-sectional study that included 1094 Spanish individuals, WHtR, AVI, and BRI were significantly associated with asymptomatic hyperuricemia after multivariate adjustment.

Obesity and hyperuricemia are two prevalent metabolic disorders that have gained substantial attention in recent years due to their significant impact on public health [47,48]. Both conditions are closely interconnected and share complex pathophysiological mechanisms, leading to a bidirectional relationship [49]. The rising prevalence of obesity worldwide has contributed to an alarming increase in the incidence of hyperuricemia, while hyperuricemia, in turn, has been linked to the development and progression of obesity-related complications [50]. Excess adipose tissue in obesity leads to the disruption of various metabolic processes, including dysregulation of substantial amounts of adipocytokines causing hyperinsulinemia [51], which increases sodium and acid uric reabsorption in the renal tubules, thereby reducing urinary excretion and leading to hyperuricemia [52]. Conversely, hyperuricemia is believed to contribute to the development and progression of obesity-related complications. Elevated serum uric acid levels have been shown to promote adipocyte dysfunction, leading to increased lipid accumulation and impaired adipogenesis [53]. Additionally, uric acid has been implicated in activating inflammatory pathways, oxidative stress, and endothelial dysfunction, all of which are key players in the pathogenesis of obesity-related comorbidities [1].

The most relevant findings of our study were that all the anthropometric indices studied, including novel and traditional, were related to asymptomatic hyperuricemia presence in the univariate analysis. However, after including the effect of potential confounders in multivariate analyses, only WHtR, AVI, and BRI were independently associated with hyperuricemia. These also provided the largest AUC in the ROC analysis. BRI combines height and waist circumference and was developed to estimate the body shape as an ellipse or oval [35]. It is considered a good predictor of metabolic syndrome, being superior to BMI [27]. On the other hand, AVI indirectly shows visceral adiposity content by evaluating the entire abdominal volume and is a good predictor for metabolic syndrome [33]. Currently, four studies have evaluated the association of any of the new anthropometric indices with hyperuricemia [39,40,41,42]. None of the studies have assessed this association using AVI, CUN-BAE, and WWI. Zhang N et al. [39], after adjusting for confounding variables, showed a significant association between ABSI, BRI, BMI, WC, and WHtR with hyperuricemia. In addition, the authors indicated that BRI, rather than ABSI, presented a stronger predictive capacity to detect hyperuricemia than BMI in females and showed similar abilities for identifying the relationship between hyperuricemia and obesity compared to WHtR and WC in females but not in males. On the other hand, Liu XZ et al. [40] found no association between ABSI, BAI, BRI, CI, or VAI and increased uric acid levels after controlling for confounding variables. However, they observed a positive association between hyperuricemia and the lipid accumulation product index (LAP) and cardiometabolic index (CMI). These indices are calculated by correlating some traditional anthropometric measures with serum lipid levels [54,55]. Whang H et al. [41] observed a significant increase in the risk of hyperuricemia associated with increased LAP, CIM, and BAI values. Finally, Tian S et al. [42] reported that the predictors BRI and WHtR were superior to ABSI, BMI, WC, or WHR for identifying hyperuricemia in women. In contrast, BMI was the anthropometric measure that provided the largest AUC in men.

Several limitations of this study should be considered. First, due to the cross-sectional design, our results only suggest association and not causality. In addition, all participants were selected from a single hospital in a specific region of Spain. Consequently, the findings may not be generalizable to other populations and ethnicities. Moreover, the serum uric acid levels were based on an evaluation of a single blood sample so some bias may have been introduced. On the other hand, our study did not assess dietary intake and physical activity levels, and some authors suggest that they affect uric acid levels. Finally, neither blood urea nitrogen nor creatinine were measured, both of which are considered risk factors for the development of hyperuricemia. However, this study is the first to assess the association between new anthropometric indices and hyperuricemia in the Spanish population. All published studies were performed on the Chinese population [39,40,41,42]. Moreover, this paper uniquely provides the optimal cut-off values of new anthropometric indices to detect hyperuricemia. It is the first study analyzing the possible relationship of the anthropometric indices AVI, CUNBAE, and WWI with the risk of hyperuricemia.

In conclusion, the results of this cross-sectional study showed that WHtR, AVI, and BRI were independently positively associated with asymptomatic hyperuricemia and could be good predictors. Combinations of various anthropometric measures could improve the prediction of metabolic diseases. We suggest that its use should be incorporated into daily clinical practice for the prevention and control of metabolic diseases, including hyperuricemia. In the future, randomized controlled studies will be necessary to assess whether modifications in anthropometric indices affect uric acid levels.

## Figures and Tables

**Table 1:** Baseline characteristics among patients according to serum uric acid levels.

	Normouricemia (*n* = 862)	Asymptomatic Hyperuricemia (*n* = 232)	*p*-Value
Age (years)	53.62 ± 12.61	59.04 ± 12.59	<0.001
Gender-males (%)	518 (60.1%)	143 (61.6%)	0.669
CV Risk factors			
Current smokers (%)	164 (19.0%)	31 (13.4%)	0.047
Hypertension (%)	491 (57.0%)	183 (78.9%)	<0.001
Dyslipidemia (%)	749 (86.9%)	194 (83.6%)	0.201
Diabetes (%)	236 (27.4%)	84 (36.2%)	0.009
Obesity (%)	312 (36.2%)	115 (49.6%)	<0.001
Metabolic Syndrome (%)	274 (31.8%)	117 (50.4%)	<0.001
Sedentary (%)	278 (32.3%)	90 (38.8%)	0.062
CV event (%)	191 (22.2%)	71 (30.6%)	0.008
Clinical and laboratory evaluation			
SBP (mmHg)	138.22 ± 17.74	141.99 ± 18.84	0.005
DBP (mmHg)	81.41 ± 10.04	80.00 ± 11.13	0.063
PP (mmHg)	56.80 ± 16.39	61.99 ± 18.53	<0.001
Total cholesterol (mg/dL)	177.96 ± 41.86	170.22 ± 39.24	0.011
LDL (mg/dL)	99.40 ± 36.56	91.73 ± 34.05	0.003
Triglyceride (mg/dL)	137.86 ± 95.47	164.89 ± 100.66	<0.001
FPG (mg/dL)	107.45 ± 28.15	116.53 ± 40.38	0.001
HbA1C (%)	6.01 ± 0.99	6.16 ± 0.94	0.035
Drugs			
Antihypertensive drugs (%)	453 (53.6%)	174 (75.0%)	<0.001
Lipid-lowering drugs (%)	667 (77.3%)	169 (72.8%)	0.149
Antidiabetic drugs (%)	218 (25.3%)	79 (34.1%)	0.008
Traditional anthropometric indices			
BMI (kg/m^2^)	29.03 ± 4.96	30.68 ± 5.01	<0.001
WHR	0.93 ± 0.09	0.96 ± 0.07	<0.001
WHtR	0.60 ± 0.08	0.64 ± 0.08	<0.001
Novel anthropometric indices			
ABSI	0.082 ± 0.008	0.084 ± 0.007	0.001
AVI	20.17 ± 5.83	22.41 ± 5.48	<0.001
BAI	32.45 ± 6.41	34.35 ± 7.97	0.001
BRI	5.62 ± 2.03	6.54 ± 2.22	<0.001
CI	1.31 ± 0.13	1.36 ± 0.11	<0.001
CUN-BAE	34.75 ± 7.91	36.97 ± 8.37	<0.001
WWI	12.04 ± 1.00	12.10 ± 1.17	0.496

Data expressed as mean ± standard deviation and frequencies (%). Abbreviations: ABSI, a body shape index; AVI, abdominal volume index; BAI, body adiposity index; BMI, body mass index; BRI, body roundness index; CAD, coronary arterial disease; CI, conicity index; CUN-BAE, Clínica Universidad de Navarra-Body Adiposity Estimator; CV, cardiovascular; DBP, diastolic blood pressure; FPG, fast plasma glucose; HbA1c, glycated hemoglobin;LDL, low-density lipoprotein; PP, pulse pressure; SBP, systolic blood pressure; WHR, waist-to-hip ratio; WHtR, waist-to-height ratio; WWI, weight-adjusted waist index.

**Table 2:** Correlation and multiple linear regression analysis between serum uric acid levels and anthropometric indices.

	Correlation Analysis		Multiple Linear Regression Analysis				
	R	*p*-Value	Model R^2^	Model Adjusted R^2^	Standardized β	*t*	*p*-Value
Traditional anthropometric indices							
BMI (kg/m^2^)	0.209	<0.001	0.189	0.181	0.136	4.12	<0.001
WHR	0.282	<0.001	0.171	0.163	0.092	2.60	0.009
WHtR	0.195	<0.001	0.179	0.171	0.136	4.18	<0.001
Novel anthropometric indices							
ABSI	0.112	<0.001	0.166	0.157	−0.003	−0.055	0.649
AVI	0.274	<0.001	0.182	0.174	0.147	4.59	<0.001
BAI	−0.055	0.040	0.141	0.163	0.098	2.68	0.007
BRI	0.188	<0.001	0.181	0.173	0.141	4.40	<0.001
CI	0.174	<0.001	0.167	0.159	0.040	1.22	0.221
CUN-BAE	−0.062	0.042	0.188	0.180	0.237	5.35	<0.001
WWI	−0.222	<0.001	0.169	0.161	−0.083	−2.19	0.029

Multiple linear regression analysis adjusted by age, sex, SBP, DBP, PP, total cholesterol, HDL, LDL, triglycerides, FPG, and HbA1C. Abbreviations: ABSI, a body shape index; AVI, abdominal volume index; BAI, body adiposity index; BMI, body mass index; BRI, body roundness index; CI, conicity index; CUN-BAE, Clínica Universidad de Navarra-Body Adiposity Estimator; WHR, waist-to-hip ratio; WHtR, waist-to-height ratio; WWI, weight-adjusted waist index.

**Table 3:** Predictors of asymptomatic hyperuricemia. Univariate analysis.

	Subjects with Hyperuricemia (*n* = 232) OR (CI%95)	*p*-Value
Age (years) ≥ 65	1.77 (1.28–2.44)	<0.001
Males (%)	1.06 (0.79–1.43)	0.669
Non-smokers	1.13 (0.83–1.54)	0.434
Current Smokers (%)	0.65 (0.43–0.99)	0.047
Ex-smokers (%)	1.12 (0.84–1.50)	0.420
Hypertension (%)	2.82 (2.00–3.97)	<0.001
Dyslipidemia (%)	0.77 (0.51–1.14)	0.201
Diabetes (%)	1.50 (1.10–2.04)	0.009
Sedentary (%)	1.33 (0.98–1.79)	0.062
Metabolic Syndrome (%)	2.18 (1.62–2.93)	<0.001
CV event (%)	1.54 (1.12–2.13)	0.008
SBP (mmHg) ≥ 140	1.43 (1.07–1.92)	0.015
DBP (mmHg) ≥ 90	0.90 (0.63–1.27)	0.566
PP (mmHg) ≥ 60	1.84 (1.39–2.49)	<0.001
TC (mg/dL) ≥ 190	0.70 (0.50–0.96)	0.031
LDL (mg/dL) ≥ 100	0.70 (0.52–0.95)	0.024
Triglyceride (mg/dL) ≥ 200	1.57 (1.09–2.25)	0.014
FPG (mg/dL) ≥ 126	0.67 (0.47–0.95)	0.028
HbA1C (%) ≥ 6.5	1.31 (0.92–1.87)	0.123
Antihypertensive drugs (%)	2.70 (1.95–3.75)	<0.001
Lipid-lowering drugs (%)	0.78 (0.56–1.09)	0.149
Antidiabetic drugs (%)	1.52 (1.11–2.08)	0.008

Abbreviations: CV, cardiovascular; DBP, diastolic blood pressure; FPG, fast plasma glucose; HbA1c, glycated hemoglobin; HDL, high-density lipoprotein; LDL, low-density lipoprotein; PP, pulse pressure; SBP, systolic blood pressure; TC, total cholesterol.

**Table 4:** Anthropometric indices as predictors of asymptomatic hyperuricemia. Univariate and multivariable analysis.

	Univariate		Multivariable	
	OR (CI%95)	*p*-Value	aOR (CI%95)	*p*-Value
Traditional anthropometric indices				
BMI ≥ 30 kg/m^2^	1.73 (1.29–2.32)	<0.001	1.32 (0.96–1.82)	0.084
WHR > 0.85 in women or 0.94 in men	1.97 (1.37–2.81)	<0.001	1.36 (0.92–2.01)	0.113
WHtR > 0.5	5.19 (1.87–14.37)	0.002	2.93 (1.03–8.37)	0.044
Novel anthropometric indices				
ABSI ≥ 0.086	1.58 (1.15–2.17)	0.005	1.15 (0.81–1.64)	0.412
AVI ≥ 23.85	1.95 (1.43–2.67)	<0.001	1.46 (1.04–2.04)	0.026
BAI ≥ 36.23	1.44 (1.04–1.98)	0.026	1.13 (0.79–1.60)	0.491
BRI ≥ 6.92	2.23 (1.63–3.04)	<0.001	1.66 (1.19–2.32)	0.003
CI ≥ 1.39	1.88 (1.39–2.55)	<0.001	1.28 (0.91–1.81)	0.150
CUN-BAE ≥ 41.07	1.43 1.03–1.97	0.028	1.24 (0.88–1.76)	0.208
WWI ≥ 12.72	1.39 (1.01–1.92)	0043	1.29 (0.90–1.83)	0.155

Multivariate analysis was adjusted by age ≥ 65 years, current smokers, hypertension presence, diabetes presence, metabolic syndrome presence, previous CV event, SBP ≥ 140 mmHg, PP ≥ 60 mmHg, TC ≥ 190 mg/dL, LDL ≥ 100 mg/dL, Triglyceride ≥ 200 mg/dL, FPG ≥ 126 mg/dL, antihypertensive, and antidiabetic drugs. Abbreviations: ABSI, a body shape index; AVI, abdominal volume index; aOR, adjusted odds ratio; BAI, body adiposity index; BMI, body mass index; BRI, body roundness index; CI, conicity index; CUN-BAE, Clínica Universidad de Navarra-Body Adiposity Estimator; OR, odds ratio; WHR, waist-to-hip ratio; WHtR, waist-to-height ratio; WWI, weight-adjusted waist index.

**Table 5:** The AUCs and optimal cut-off values of anthropometric indices for identifying asymptomatic hyperuricemia.

	AUC (95%IC)	*p*-Value	Sensitivity	Specificity	Youden’s Index	Cut-Off
Traditional anthropometric indices						
BMI	0.600 (0.560–0.640)	<0.001	0.659	0.523	1.182	28.63
WHR	0.582 (0.542–0.622)	<0.001	0.737	0.387	1.124	0.91
WHTR	0.624 (0.582–0.664)	<0.001	0.780	0.411	1.192	0.57
Novel anthropometric indices						
ABSI	0.579 (0.539–0.619)	<0.001	0.496	0.635	1.131	0.083
AVI	0.621 (0.582–0.660)	<0.001	0.685	0.515	1.200	19.55
BAI	0.555 (0.513–0.598)	0.009	0.263	0.852	1.115	38.90
BRI	0.624 (0.585–0.664)	<0.001	0.556	0.635	1.191	5.97
CI	0.611 (0.571–0.650)	<0.001	0.672	0.505	1.178	1.31
CUNBAE	0.570 (0.529–0.612)	0.001	0.224	0.889	1.113	44.87
WWI	0.503 (0.459–0.547)	0.904	0.315	0.752	1.067	12.68

Abbreviations: ABSI, a body shape index; AVI, abdominal volume index; AUC, area under curve; BAI, body adiposity index; BMI, body mass index; BRI, body roundness index; CI, conicity index; CUN-BAE, Clínica Universidad de Navarra-Body Adiposity Estimator; WHR, waist-to-hip ratio; WHtR, waist-to-height ratio; WWI, weight-adjusted waist index.

## Data Availability

The data are available from the corresponding author.
